# EHMTI-0015. Impact of headache-attributed burden on productivity and quality of life in russia: structured healthcare is urgently needed

**DOI:** 10.1186/1129-2377-15-S1-B3

**Published:** 2014-09-18

**Authors:** I Ayzenberg, Z Katsarava, V Osipova, G Tabeeva, M Chernysh, TJ Steiner

**Affiliations:** 1 Neurology, Ruhr University Bochum St.Josef Hospital, Dortmund, Germany; 2 Neurology, Evangelic Hospital Unna / University of Duisburg-Essen, Unna, Germany; 3 Neurology, I.M. Sechenov First Moscow State Medical University, Moscow, Russia; 4 Sociology, Insitute of Sociology, Moscow, Russia; 5 Neurology, Norwegian University of Science and Technology, Trondheim, Norway

## Introduction

Tension-type headache (TTH) and migraine are common, respectively the second and third most prevalent disorders globally. Their direct burdens of recurrent pain and associated symptoms are damaging to quality of life (QoL) and disabling – migraine being the seventh-highest specific cause of disability worldwide.

## Aims

The study evaluated headache-attributed burden and its impact on productivity and quality of life in Russia. Its purpose was to support recommendations for change.

## Methods

A countrywide population-based random sample of 2725 biologically unrelated adults in 35 cities and nine rural areas of Russia were interviewed in a door-to-door survey. The structured questionnaire enquired into symptom burden, functional disability, lost productive time and QoL and willingness to pay (WTP) for adequate headache treatment.

## Results

Mean lost paid-work days in the previous 3 months were 1.9±4.2, and mean lost household work days 3.4±5.7. The estimated annual indirect cost of primary headache disorders was USD 22.8 billion, accounting for 1.75% of gross domestic product. QoL was reduced by all types of primary headaches. According to WHOQoL-8, it was significantly lower in those with headache on ≥15days/month than in those with episodic headache (24.7±4.6 vs. 28.1±5.0; P<0.05) and lower in those with migraine than in those with TTH (27.1±4.9 vs. 28.8±5.0; P<0.05). Average WTP was sufficient for adequate headache treatment and correlated with illness severity.

## Conclusions

Headache is common, burdensome and costly in Russia and, manifestly, poorly mitigated by existing healthcare. Structured healthcare services for headache need to be urgently put in place.

No conflict of interest.

